# The mediating effect of critical thinking between interest in learning and caring among nursing students: a cross-sectional study

**DOI:** 10.1186/s12912-023-01181-4

**Published:** 2023-02-03

**Authors:** Lai Kun Tong, Mio Leng Au, Yue Yi Li, Wai I. Ng, Si Chen Wang

**Affiliations:** Kiang Wu Nursing College of Macau, Macau, China

**Keywords:** Caring, Critical thinking, Interest in learning, Nursing student

## Abstract

**Background:**

Previous studies have explored the influence of interest in learning on caring and critical thinking, as well as the relationship between caring and critical thinking. However, the mediating effect of critical thinking in interest learning and caring among nursing students has not been clarified.

**Methods:**

Nursing students who enrolled for the 2021/2022 academic year in diploma, undergraduate, or graduate programs in five provinces of China (Guangdong, Sichuan, Jiangsu, Hunan and Macao). An online survey with a convenience sampling method was employed to collect data. The questionnaires were administered to 692 participants between January 20 and 26, 2022. Amos 26.0 was employed to establish the structural equation modelling and analyze the mediating effect of critical thinking on interest in learning and caring.

**Results:**

The first regression equation showed that interest in learning significantly influenced caring (β = 0.339, *p* <  0.001). The third regression equation showed that critical thinking significantly influenced caring (β = 0.494, *p* <  0.001). The effect of interest in learning on caring was less in the third equation than in the first equation (β = 0.154 vs β = 0.339), which indicates partial mediation. Furthermore, interest in learning had an indirect positive effect (β = 0.186, *p* <  0.001) on caring mediated by critical thinking, with 95% confidence interval of 0.142 to 0.233.

**Conclusions:**

Critical thinking was a significant mediator of the relationship between interest in learning and caring. It is suggested that nursing colleges and instructors should take into account students’ interest in learning and critical thinking as potential intervention elements to enhance caring.

## Background

Nursing students must possess highly specialized competencies to accurately assess the state of patients and cope with potential problems. The requirements for nursing students vary from region to region, but there are some commonalities, as caring and critical thinking were identified as essential competencies [1]. Caring is the core of nursing practice, caring behavior varies in different societies and cultures [2]. Caring in nursing involves respect, support, and meeting patients’ needs [3]. The implementation of caring in clinical practice can not only benefit patients’ physical and mental health [4], but also improve nurse-patient relationships and enhance patients’ satisfaction [5]. Nurses who are trained in caring display greater problem-solving skills [6], are more satisfied in their jobs [4], and have a stronger sense of professional identity [6].

Critical thinking is closely related to nursing competence [7] and patient outcomes [8]. The undergraduate nursing curriculum and the core competencies of registered nurses emphasize critical thinking [9, 10]. To practice caring, nurse involve providing personalized nursing in response to the patients’ needs, which requires critical thinking skills [11]. Research on caring and critical thinking is not only concerned with concepts, influencing factors, but also explores the relationship between them [12, 13]. Researchers have found that caring and critical thinking are positively related both in western and oriental nursing students [11, 12]. Caring and critical thinking skills are not inherent, but can be developed. Nursing educators use a variety of teaching methods to foster caring and critical thinking in nursing students [14, 15]. Learning outcome is affected by interest in learning [16]. Learning is a process influenced by interest [17], and interest in learning will have a major impact on the way students learn [18]. The greater the interest of students, the better the learning outcomes will be [16]. The following conceptual framework was established based on the previous studies: 1) interest in learning is positively correlated with critical thinking, 2) interest in learning is positively correlated with caring, 3) critical thinking is positively correlated with caring, and 4) critical thinking is a mediator between interest in learning and caring.

Previous studies have explored the influence of interest in learning on caring and critical thinking, as well as the relationship between caring and critical thinking. However, the mediating effect of critical thinking in interest learning and caring among nursing students has not been clarified. The objectives of this study were to: 1) describe caring among nursing students in China and 2) examine the relationships among interest in learning, caring, and critical thinking.

## Methods

The study followed the STROBE reporting guideline [19].

### Study design

A descriptive cross-sectional study was conducted between January 20 and 26, 2022 to examine the relationships among interest in learning, caring, and critical thinking among nursing students in China.

### Settings and participants

The participants were nursing students from five provinces in China. The inclusion criteria were nursing students who enrolled in diploma, undergraduate or graduate programs in Guangdong, Sichuan, Jiangsu, Hunan and Macao in the 2021/2022 academic year and volunteered to participate. The exclusion criteria were nursing students who could not read or write Chinese. Due to the limited information available, it was assumed that the effect of interest in learning on critical thinking and the effect of critical thinking on caring would be small, which results in maximum variability. According to the empirical power tables, a sample size of 558 or greater is required for 0.8 power when using the percentile bootstrap to test the mediating effect [20].

### Procedures

A convenience sampling method was employed. The data were collected during the COVID-19 pandemic, the research team used the most widely used social media platform in China, Wechat, to recruit and promote the study. Participants scanned the QR code of the poster for access to the online survey platform. The participants read and agreed to the informed consent before starting to fill in the questionnaire.

### Measures

#### Caring dimensions inventory

The Caring Dimensions Inventory consisted of 25 items, each measured on a 5-point Likert scale ranging from 1 (strongly disagree) to 5 (strongly agree) [21]. Scores ranged from 25 to 125 points, with higher scores indicating a higher level of caring. The Cronbach’s α coefficient and content validity index of the Chinese version were 0.97 and 0.98 respectively [22].

#### Study interest questionnaire

Study Interest Questionnaire contained 18 items rated on a 4-point Likert scale [23]. Scores ranged from 1 to 4 points, with higher scores indicating a higher level of interest in learning. It demonstrated good reliability (Cronbach’s α coefficient = 0.90) [23].

#### Yoon’s critical thinking disposition

Yoon’s Critical Thinking Disposition consisted of 27 items scored on a 5-point Likert scale [24]. Scores ranged from 1 to 5 points, with higher scores indicating a higher level of critical thinking. It exhibited acceptable reliability (Cronbach’s α coefficient = 0.84) [24].

### Ethical considerations

The Research Management and Development Department at the authors’ college provided ethical approval for the study (ethical approval number: REC-2021.801). All methods were performed in accordance with the relevant guidelines and regulations. The survey was conducted anonymously. Participants could withdraw from participation at any time without any loss of benefits.

### Data analysis

SPSS 26.0 was used to conduct descriptive statistics on the characteristics of participants and Pearson correlation analysis on the correlation between interest learning, caring and critical thinking. A *p*-value ≤0.05 was considered statistically significant.

Amos 26.0 was employed to establish the structural equation modelling and analyze the mediating effect of critical thinking on interest in learning and caring. The two-sided 95% bootstrap percentile confidence intervals were computed using 2000 replications to validate the model. A 95% confidence interval without zero indicates statistical significance.

## Results

### Participants’ characteristics

A total of 692 valid questionnaires were collected, with the majority of participants being female (*n* = 618, 89.3%) and an average age of 22.0. Most of the participants were enrolled in undergraduate nursing programs (*n* = 369, 53.3%) at schools mainly located in Jiangsu Province (*n* = 200, 28.9%) (Table 1).

**Table 1:** Characteristics of participants (*n* = 692)

Characteristics	Categories	*n* (%)	Mean (Standard deviation)
Gender	Female	618 (89.3)	
	Male	74 (10.7)	
Age (years)			22.0 (4.3)
Degree course	Diploma	191 (27.6)	
	Undergraduate	369 (53.3)	
	Graduate	132 (19.1)	
School location	Guangdong Province	131 (18.9)	
	Sichuan Province	150 (21.7)	
	Jiangsu Province	200 (28.9)	
	Hunan Province	120 (17.3)	
	Macao	91 (13.2)	

### Levels of caring, interest in learning, and critical thinking

The mean score was 103.62 out of 125 for caring, 2.78 out of 4 for interest in learning, and 3.65 out of 5 for critical thinking (Table 2).

**Table 2:** Descriptive statistics for caring, interest in learning, and critical thinking (*n* = 692)

Variables	Mean	Standard deviation	Participant scoring	
			Minimum	Maximum
Caring	103.62	14.01	63.00	125.00
Interest in learning	2.78	0.36	1.17	4.00
Critical thinking	3.65	0.46	1.48	5.00

### Relationships among interest in learning, caring, and critical thinking

The correlations among caring, interest in learning, and critical thinking are shown in Table 3. Interest in learning was positively correlated with critical thinking (*r* = 0.376, *p* <  0.01). Interest in learning was positively correlated with caring (*r* = 0.339, *p* <  0.01). Critical thinking was positively correlated with caring (*r* = 0.551, *p* <  0.01).

**Table 3:** Correlations among caring, interest in learning, and critical thinking (*n* = 692)

Variables	Caring	Interest in learning	Critical thinking
Caring	1		
Interest in learning	0.339**	1	
Critical thinking	0.551**	0.376**	1

Note. ⁎⁎ *p* < 0.01 (2-tails)

### Regression analysis

The results of linear regression analyses on the mediating effect of critical thinking between interest in learning and caring, are shown in Table 4. The first regression equation showed that interest in learning significantly influenced caring (β = 0.339, *p* <  0.001). The second regression equation showed that interest in learning significantly influenced critical thinking (β = 0.376, *p* <  0.001). The third regression equation showed that critical thinking significantly influenced caring (β = 0.494, *p* <  0.001). The effect of interest in learning on caring was less in the third equation than in the first equation (β = 0.154 vs β = 0.339), which indicates partial mediation.

**Table 4:** The results of regression analyses

Dependent variable	Independent variable	β	t	F	Adjusted R^2^
Caring	Interest in learning	0.339	9.479***	89.852***	0.114
Critical thinking	Interest in learning	0.376	10.664***	113.711***	0.140
Caring	Interest in learning	0.154	4.551***	165.352***	0.322
	Critical thinking	0.494	14.602***		

Note. ⁎⁎⁎ *p* < 0.001 (2-tails)

### Mediating effect of critical thinking between interest in learning and caring

**Fig. 1:**
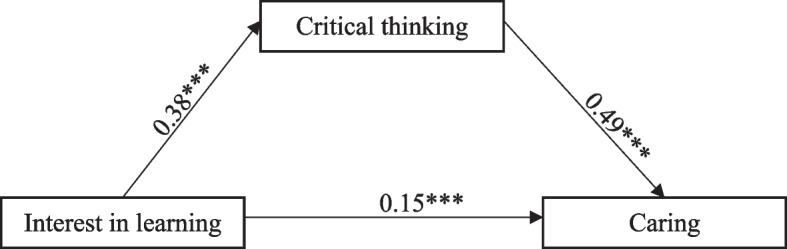
Mediation model of effect of critical thinking between interest in learning and caring. Note. ⁎⁎⁎ *p* < 0.001 (2-tails)

Table 5 and Fig. 1 illustrate the mediating effects of critical thinking. Critical thinking was positively related to caring (β = 0.49, *p* <  0.001). Interest in learning was positively correlated with critical thinking (β = 0.38, *p* < 0.001). Moreover, interest in learning had an indirect positive effect (β = 0.49 × 0.38 = 0.186, *p* < 0.001) on caring mediated by critical thinking, with 95% confidence interval of 0.142 to 0.233. As this confidence interval did not contain zero, it was concluded that critical thinking was a significant mediator of the relationship between interest in learning and caring. The direct positive effect of interest in learning on caring was 0.15 (*p* < 0.001), so the mediating effect of critical thinking was partial. The total effect of interest in learning on caring was 0.339.

**Table 5:** Mediating effect of critical thinking between interest in learning and caring (*n* = 692)

	Estimate	95% CI	*p*
Indirect effect			
Interest in learning → Critical thinking → Caring	0.186	0.142 - 0.233	< 0.001
Direct effect			
Interest in learning → Critical thinking	0.376	0.303 – 0.448	< 0.001
Interest in learning → Caring	0.154	0.089 - 0.215	< 0.001
Critical thinking → Caring	0.494	0.432 – 0.556	< 0.001
Total effect			
Interest in learning → Caring	0.339	0.279 - 0.399	< 0.001

## Discussion

This study aimed to examine caring among nursing students in China and the relationships among interest in learning, caring, and critical thinking. Findings of this study generally supported the conceptual framework proposed in it. The results of the mediation analysis confirmed that critical thinking played a partially mediating role between interest in learning and caring among nursing students. The findings of regression showed that interest in learning had a significant impact on caring, which was similar to the results of previous studies [25–27]. It is suggested that increasing nursing students’ interest in learning can be beneficial to their caring. Caring is not instinctive, but gradually grows and develops through learning and practice under the influence of both environment and education [28, 29]. The evidence is clear that students learn more when they are interested in the course [30, 31]. However, several studies have shown that nursing students’ caring does not change throughout their educational journey, and some studies even reported a decline [32, 33]. The two main components of caring are instrumental and expressive. Instrumental component is devoted to meeting physical and technical aspects of patients’ care, while expressive component involves meeting their psychological, social, and emotional needs [34, 35]. The existing educational system effectively promotes instrumental caring, while expressive caring calls for more efficient strategies [36, 37]. Critical thinking helps nursing students to perform expressive caring [38]. Nursing students who can demonstrate critical thinking have the ability to seek and examine information, and integrate information together to synthesize appropriate solutions [39]. Caring must begin by understanding the needs of patients, so that each patient can receive appropriate support according to their unique personalities [40]. Nursing students should use critical thinking in their practice of caring. Therefore, the effect of nursing students’ interest in learning on increasing caring maybe enhanced by strengthened their critical thinking.

The findings of this study revealed that interest in learning had an indirect positive effect on caring mediated by critical thinking. During this process, how does critical thinking come into play? Caring is the combination of purposeful nursing acts and attitudes designed to alleviate patients’ discomfort and meet their needs [41]. As part of the implementation of caring, it is imperative to collect and integrate all aspects of patient information in order to fully assess their condition and provide intervention accordingly [42]. Making decisions based on analysis and evaluation of problems requires critical thinking [43]. Critical thinking in clinical setting involves problem solving, caring, unbiased inquiry, intuition, and reflection in action [44]. Critical thinking enables both implicit and explicit information about the patient to be considered, as well as the integration of all useful information, so that caring can be accomplished. A positive correlation has been found between critical thinking and caring in previous studies [12]. Despite the fact that critical thinking cannot guarantee the practice of caring, it makes it possible to do so. Therefore, it is important to develop critical thinking among nursing students in order to cultivate their caring.

It was demonstrated that interest in learning was an important antecedent variable for nursing students to improve caring through critical thinking. The average score of critical thinking for nursing students in China was higher than the median in this study, indicating their critical thinking was at a medium level, but slightly lower than that of South Korean nursing students using the same instrument [45]. It is therefore suggested that courses focusing on caring should also introduce critical thinking as one of their learning outcomes in an effort to develop nursing students’ caring skills. The mediating role of critical thinking offers a new perspective for improving nursing students’ caring.

It adds to the literature on nurturing nursing students’ caring, but there are some limitations to the study. First, the findings were based on cross-sectional data, making it difficult to verify the causal relationship between variables. Future studies should employ longitudinal or experimental designs to clarify the relationship between these variables in more detail. Second, this study relied on self-reported questionnaires, resulting in an inevitable bias in information. To remedy this shortcoming, future research should focus on obtaining data using external tools such as classroom observations. Third, this study was conducted during the COVID-19 pandemic, which has significant implications for nursing students and nursing education. The results from this study should be interpreted with care once the pandemic has ended.

## Conclusion

This study explored the little-studied topic of critical thinking and caring in nursing students, enriching the literature concerning nursing education. This study found that there was a significant relationship between interest in learning, critical thinking and caring. Importantly, this study revealed that students’ critical thinking played a mediating role in the relationship between interest in learning and caring, indicating that critical thinking may explain the relationship between interest in learning and caring.

## Data Availability

The data that support the findings of this study are available from the corresponding author, upon reasonable request.
